# Retraction: A novel observation of pubic osteomyelitis due to *Streptococcus viridans *after dental extraction: a case report

**DOI:** 10.1186/1752-1947-3-122

**Published:** 2009-11-13

**Authors:** Naseem Naqvi, Rizwana Naqvi, Christopher Wong, Sushmita Pearce

**Affiliations:** 1 Department of Acute Medicine, Dumfries & Galloway Royal Infirmary, UK; 2 Department of Medicine, Macclesfield District General Hospital, East Cheshire NHS Trust, UK; 3 Department of Renal Medicine, Aintree University Hospitals NHS Trust, Liverpool, UK; 4 Department of Medicine, Royal Albert Edward Infirmary, Wigan, UK

## Retraction

BioMed Central is publishing a retraction of this article [1] because the article was published in error. The case report contained erroneous details of the patient's presentation and not all original authors agreed to publish. SP had no involvement whatsoever with this article and was never consulted prior to its publication. BioMed Central apologize to the readers for the error, and to the authors for any inconvenience caused.

## References

[B1] Naqvi N Naqvi R Wong C Pearce S A novel observation of pubic osteomyelitis due to *Streptococcus viridans *after dental extraction: a case report Journal of Medical Case Reports 2008 2 255 10.1186/1752-1947-2-255 18671843 PMC2517070

